# Feline noroviruses: investigation of epidemic potential and genetic/structural insights of host-based separation and recombination

**DOI:** 10.1128/spectrum.01662-26

**Published:** 2026-09-02

**Authors:** Ákos Boros, Akari Sugihara, Benigna Balázs, Fruzsina Tóth, Zoltán Lajos, Péter Pankovics, Péter Urbán, Róbert Herczeg, Gábor Reuter

**Affiliations:** 1 Department of Medical Microbiology, Medical School, University of Pécs 37656, Pécs, Hungary; 2 Duo-Bakt Veterinary Microbiology Laboratory, Veresegyház, Hungary; 3 Szentágothai Research Centre, Bioinformatics Research Group, Genomics and Bioinformatics Core Facility, University of Pécs 37657, Pécs, Hungary; The Ohio State University, Columbus, Ohio, USA

**Keywords:** norovirus, feline, enteric outbreak, phylogenetics, recombination, host adaptation, structural analysis

## Abstract

Noroviruses (family *Caliciviridae*) are important enteric pathogens in humans and animals. The closely related GIV and GVI noroviruses in cats and dogs suggest interspecies circulation; however, their diversity and host restrictions have not been fully elucidated. In this study, we retrospectively investigated noroviruses in diarrheic cats in Hungary, determined the complete genomes of representative strains (*n* = 3), and performed comparative sequence and structural analyses of all available feline and canine GIV and GVI sequences. Fecal samples (*n* = 90; November 2024–January 2025) from stray and free-roaming cats were screened using RT-PCR. Capsid sequences and complete genomes were determined using 3′/5′RACE/RT-PCR, NovaSeq+ (Illumina), and Sanger sequencing techniques. Analyses included phylogenetic trees, p-distance comparison plots, recombination detection, VP1 P-domain dimer, and complete VP1-based capsid modeling. Eight (8.9%) samples were norovirus positive. All known feline genotypes (*n* = 4 GIV.2, *n* = 1 GVI.1, *n* = 1 GVI.2) were identified, each with the (GVI.P1) P-type. Nearly identical GIV.2 strains were found in the Budapest area (November 2024), representing the first non-shelter-associated case accumulation (a potential outbreak) of norovirus in cats. One complete genome of each genotype was determined. Multiple host-specific substitutions separate feline and canine noroviruses across viral proteins, including the surface-exposed VP1 P-domains and a feline-specific 10- or 12-amino-acid-long deletion in the GVI.2 P2 subdomain of currently unknown importance. Lower p-distance boundaries for inter-/intra-genogroups and inter-P-types were also established. Recombination at the ORF1/ORF2 junction was observed in two feline lineages, indicating that recombination and host adaptation jointly drive the evolution of carnivore noroviruses.

**IMPORTANCE:**

Despite the major public health relevance of noroviruses, research has predominantly focused on human strains, while noroviruses of carnivores, particularly feline strains, remain poorly characterized. This study provides genetic, phylogenetic, and structural insights into feline noroviruses circulating in Hungary, including indications of the first reported large, non-shelter-associated potential outbreak of feline GVI.2 norovirus among free-living cats in Budapest and its surrounding areas, highlighting the natural epidemic potential of these viruses. Comparative genomic analyses demonstrated consistent host-based separation of closely related feline and canine GIV and GVI noroviruses across multiple genomic regions, supporting ongoing host-specific evolution. Structural analyses identified characteristic deletions within the highly variable surface-exposed P2 subdomain of feline GVI.2 capsid proteins, suggesting potential roles in interactions of receptors/attachment factors and/or immune evasion. Additionally, this study demonstrates the utility of pairwise p-distance analyses for recombinant strain recognition and for refining genetic demarcation criteria within feline and canine norovirus genogroups and P-types.

## INTRODUCTION

Noroviruses of the family *Caliciviridae*, genus *Norovirus*, have a positive-sense single-stranded RNA genome of approximately 7.6 kb in length. Most of them contain three overlapping open reading frames (ORFs) flanked by short 5′/3′ untranslated regions (UTRs) and a 3′ poly(A) tail. The first and largest, ORF1, encodes nonstructural proteins (NS1-7) essential for viral replication (such as conserved NS7/RNA-dependent RNA polymerase/RdRp, NS6/protease, NS3/NTPase/helicase, and NS5/VPg) and host interactions and adaptation (such as the highly variable NS1/2 and NS4/3A-like peptide) (1, 2). The second ORF (ORF2) encodes the immunodominant major capsid protein, VP1, which forms the outer shell, whereas the third ORF (ORF3) encodes a minor capsid protein, VP2, which is responsible for capsid assembly, stability, and genome packaging (3, 4)(5). The VP1 proteins of noroviruses have a conserved domain organization consisting of a shell (S) domain and a protruding (P) domain, which can be further subdivided into P1 and P2 subdomains (6). The conserved S domain forms the inner capsid shell, whereas the variable P domain, especially the most diverse P2 subdomain, is surface-exposed and contains epitopes. The P2 subdomains are also involved in the binding of host-specific receptors and attachment factors (e.g., human histo-blood group antigens) and immune evasion (7–9).

Genetically diverse noroviruses are classified into ten genogroups (GI–GX) and 49 genotypes, as well as eight P-groups and 60 P-types, based on the use of phylogenetic tree-based genetic distances in the VP1 amino acid and NS7/RdRp nucleotide sequences, respectively (10).

Due to the presence of ORF1/ORF2 recombinants, a dual-typing nomenclature for noroviruses was introduced, listing the capsid genogroup and genotype first, followed by the P-group and type in square brackets (for example, GIV.2[GVI.P1]) (10, 11).

GI, GIV, GVIII, GIX, and especially GII noroviruses are the leading causes of acute gastroenteritis in humans worldwide, frequently causing outbreaks with winter seasonality in temperate regions (12, 13). In addition to humans, noroviruses have also been detected in various vertebrate species, including carnivores within the families *Felidae* (GIV and GVI) and *Canidae* (GIV, GVI, and GVII) (13, 14). Based on the results of field and experimental infection studies, noroviruses can cause acute diarrhea and vomiting accompanied by fecal viral shedding and transient viral RNAemia, providing direct evidence of pathogenicity in domestic cats (15, 16). Outbreaks have only been reported in enclosed settings, such as an animal shelter in the USA, where GIV noroviruses were detected in 6 of 14 affected kittens (16, 17). Subsequent studies have repeatedly identified noroviruses in cats with diarrhea, particularly young animals, although there have been no reports of non-shelter-associated norovirus outbreaks in felines (15). Nevertheless, the overall clinical burden of noroviruses in cats remains uncertain because the enteric molecular prevalence is generally low (1.2%–6.2%) outside outbreaks, while serological studies indicate that exposure is more widespread (16.1%–26.2%), and relatively frequently found concurrent infections with other enteric pathogens such as feline panleukopenia virus, feline coronavirus, or feline calicivirus may also complicate causal interpretation (15, 16, 18–20).

This study used the updated classification scheme introduced by Chhabra et al. (10) for feline and canine noroviruses to address existing inconsistencies regarding GVI genotype numbers and the dual classification of certain RdRp sequences as either GIV or GVI (15, 21, 22).

Although noroviruses are generally considered to have marked host species specificity with limited cross-species transmission capabilities (14, 23), phylogenetically similar felid and canid GIV and GVI noroviruses belong to the same three genotypes (GIV.2, GVI.1, and GVI.2), which are collectively referred to as small carnivore noroviruses, suggesting interspecies circulation of these genotypes among these animals (17, 18). Although there are two known GVI P-types (GVI.P1 and P2), all three genotypes of feline noroviruses share the same (GVI.P1) P-type, indicating the possible inter-genogroup and inter-genotype recombination nature of GIV.2 and GVI.2 noroviruses, respectively (10, 14, 18).

While noroviruses have been extensively studied in humans, limited information is available on their genotype diversity, pathogenesis, and epidemic potential in carnivores, especially felines. To date, only 20 partial and 4 complete (*n* = 3 GIV.2 and *n* = 1 GVI.1) feline norovirus genomes from Japan (22), Australia (24), the USA (17), Italy (25), and additional sequences from Hungary and China with “unpublished” annotations are known. Only a single partial GVI.2 feline norovirus genome (KT245136) is available from Italy, which shows a close relationship with canine GVI.2 noroviruses, indicating interspecies transmission (18). Comprehensive sequence and structural analyses of the ORF1/NS and ORF2/VP1 sequences of feline and canine GVI and GIV noroviruses are also lacking, partly because of the limited number of available complete genomes/CDS.

The objectives of this study were to (i) retrospectively investigate the presence and genotype diversity of noroviruses in diarrheic cats in Hungary, (ii) increase the number of complete genomes of various norovirus types, and (iii) perform comparative sequence, recombination, and structural analyses of feline and closely related canine GVI and GIV noroviruses using newly acquired and publicly available GenBank sequences. Comparative analyses of the sequence, phylogenetic, and P domain dimer structures identified significant distinctions, including host-specific substitutions in all NS and VP peptides, as well as recombinant strains of feline and canine GVI and GIV noroviruses. These findings also suggest that host adaptation and recombination are important driving forces in the evolution of these carnivore noroviruses.

## RESULTS

### RT-PCR-based norovirus screening and complete ORF1 analysis of selected strains

A total of 90 fecal samples collected from diarrheic cats between November 2024 and January 2025 were retrospectively tested for the presence of noroviruses using two sets of generic primer pairs, both of which targeted the conserved ORF1^RdRp^ (Table 1).

**TABLE 1:** List and characteristic features of oligonucleotide primers used for screening and 3′ rapid amplification of cDNA ends (3′ RACE) semi-nested PCR (3-RACE-snPCR1/2) reactions. F, forward; R, reverse; Tm, melting temperature

Target (Region)	Reaction type	Oligonucleotide ID	Sequence (5′–3′)	Length (nt)	Tm (°C)	PCR product size
Norovirus (ORF1^RdRp^)	Screening RT-PCR	NoV-YGDD-Rgen	ATC TCA TCA TCA CCA TAR AAR GAG	24	60	550 bp
		NoV-KDEL-Fgen^*a*^	ACC GCA GCA CTC AAR GAY GA	20	63	
	Screening RT-PCR	JV13I (reverse)	TCA TCA TCA CCA TAG AAI GAG	21	59	281 bp
		JV12Y-RW (forward)^*b*^	RTA CCA CTA TGA TGC WGA YTA	21	57	
3′ Poly(A)-tail	3′RACE-RT	OligodT-Anchor-Adapter	GCG CGC GCC ACC AAT TTA AA T(15)V	36	>42	
	3′RACE-snPCR1/2	Adapter-1	GCG CGC GCC ACC AAT TTA AAT	21	66	3,644 bp (PCR1) 3,416 bp (PCR2)

^*a*^ Indicates forward primer of the first 3′ RACE PCR reaction.

^*b*^ Indicates forward primer used for the second snPCR reaction, both together with adapter-1 reverse primer. Note that JV12Y-RW/JV13I primers were based on the screening primers of Vennema et al. (26) with minor modifications (the JV12Y forward primer contains mixed bases of “R” and “W” at the 1st and 15th positions, respectively).

A total of 8/90 samples (8.9%) were RT-PCR-positive for norovirus using both screening primer pairs. Sanger sequencing and BLASTn analyses of the YGDD/KDEL PCR products confirmed the presence of noroviruses in the RT-PCR-positive samples. The acquired partial RdRp sequences showed >89.3% nucleotide (nt) pairwise identity to each other and 91.1%–95.6% nt identity to feline noroviruses as the most similar sequences found in the GenBank database by BLASTn analyses.

**Fig 1:**
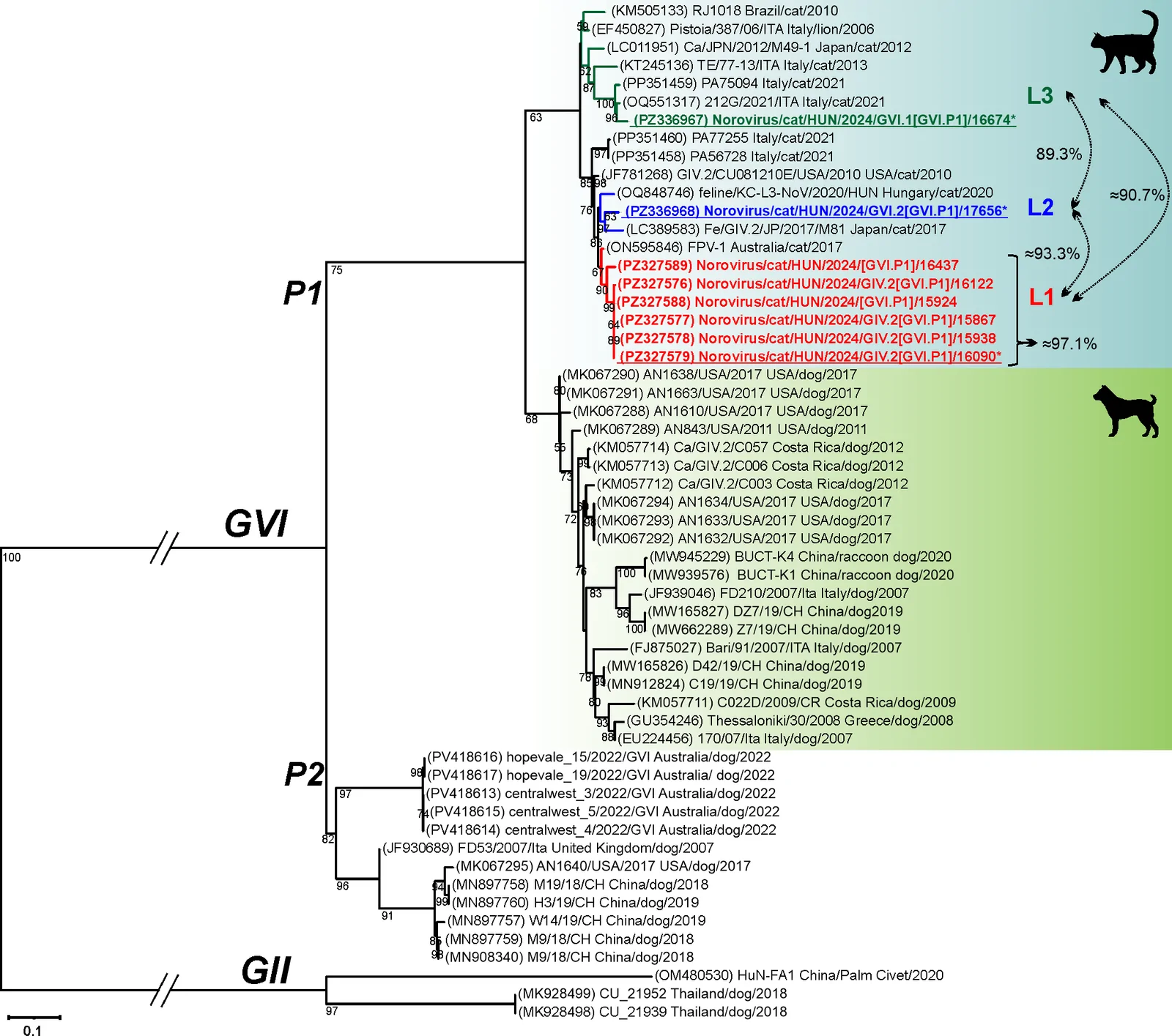
Phylogenetic analysis of partial RdRp nucleotide sequences of the study strains (marked in red, blue, and green according to the lineages L1–L3, respectively). The maximum likelihood tree (best-fit model: TNe + G4 with 1,000 ultrafast bootstrap/BS/ replicates) contains all known feline and canine GVI.P1/2 noroviruses with available corresponding sequences, together with canine GII strains, which were used as outgroups. Strains used for complete genome sequencing are marked with asterisks and underlined. Sequence names include (accession number) followed by the strain name, country/host/collection year, except for the study sequences, where the sample ID number can be found after the dual-typing designations. Feline and canine origin GVI.P1 sequences are marked with teal and green backgrounds, respectively. Pairwise nt identity values between the study strains connected by double dotted arrows can be found on the upper right side of the figure. Only BS values of 50 or more are indicated at the node. To include as many feline norovirus sequences as possible, only a 285-nt-long RdRp sequence was analyzed in this study.

The results of phylogenetic analysis indicated that all eight study RdRp sequences clustered together with feline GVI.P1 noroviruses and were separated into three different lineages (L1–L3) (Fig. 1). The six study norovirus sequences in the L1 lineage were highly similar to each other (sharing >97.1% pairwise nt identity). The remaining two study strains belong to two additional separate lineages (L2 and L3), which show >93% and >96% identity to GIV and GVI feline noroviruses, respectively. There is only ≤93.3% pairwise nt identity between members of the three lineages (Fig. 1).

**Fig 2:**
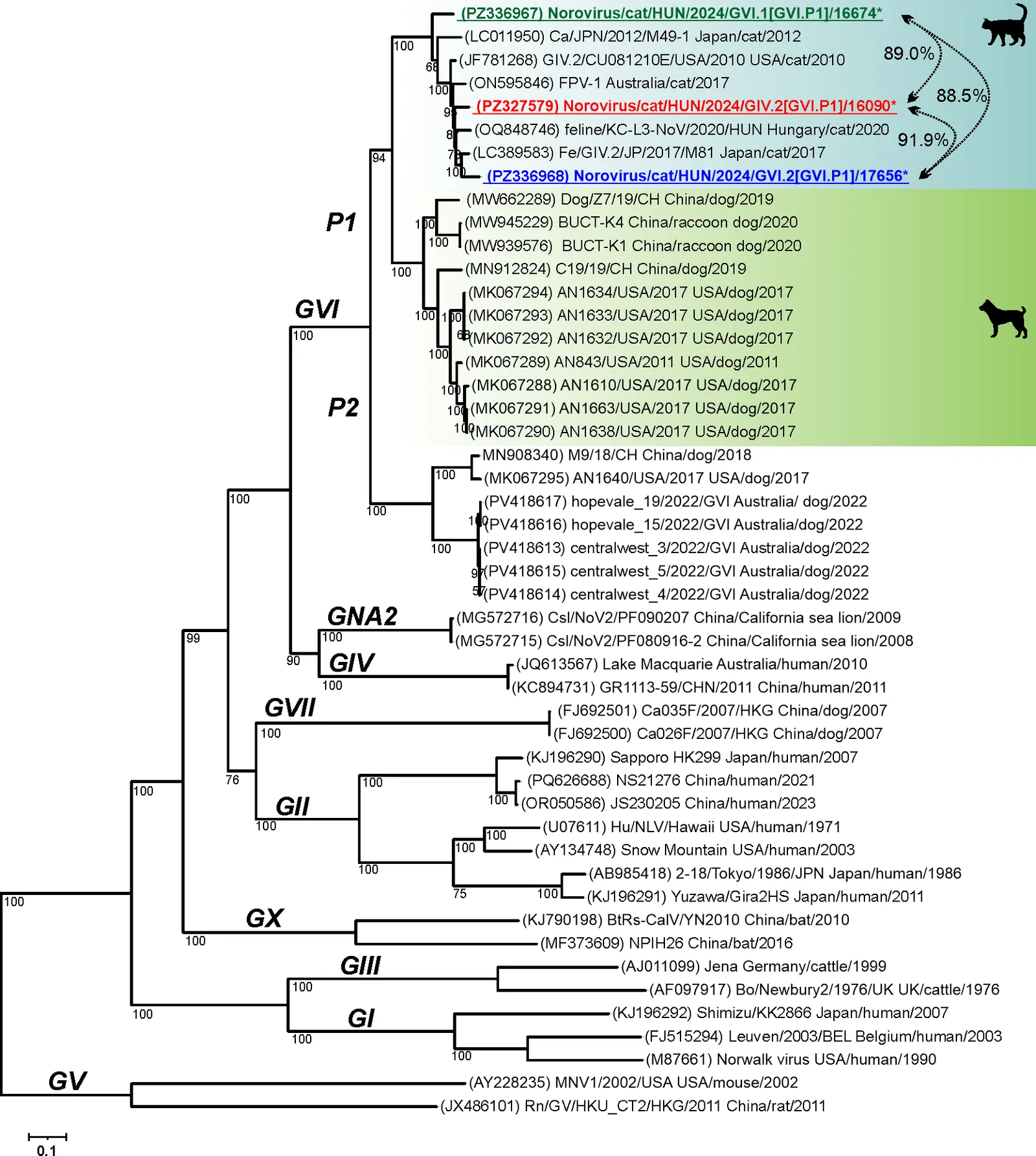
Phylogenetic analysis of complete ORF1 nucleotide sequences of the study strains (shown in red, blue, and green according to the RdRp lineages L1-L3/genotypes, respectively). The maximum likelihood tree (best-fit model: TIM2 + F + I + G4 with 1,000 ultrafast bootstrap/BS/ replicates) contains all known feline and canine noroviruses with available complete ORF1 sequences, together with representative sequences of 10 norovirus P groups (GI–GX). Sequence names include (accession number) followed by the strain name, country/host/collection year, except for the study sequences, where the sample ID number can be found after the dual-typing designations. Feline- and canine-origin GVI.P1 norovirus sequences are marked with teal and green backgrounds, respectively. Pairwise nucleotide identity values between the study strains connected by double dotted arrows can be found on the upper right side of the figure.

To analyze the complete ORF1 genome regions of all three RdRp-lineages, the c.a. 4,220-nt-long 5′ upstream genome parts (including the complete 5′UTR and ORF1 regions) of one representative member of each lineage (IDs: 16090 from L1, 17656 from L2, and 16674 from L3) were determined using RT-PCR, 5′RACE PCR methods, Sanger sequencing, and primer-walking techniques. The acquired lengths of the complete ORF1s are 5,241 nt (encoding a 1,746-aa-long viral polyprotein) for all three strains, which share up to 91.9% nt (96.1% aa) pairwise identity with each other (Fig. 2). All three strains clustered together with the cat-origin GVI.P1 noroviruses but were clearly separated from each other phylogenetically (Fig. 2).

### Determination and genetic analyses of the feline norovirus capsid sequences

To investigate sequence diversity and potential genotype/variant co-infections, the c.a. 3,600-nt-long 3′ genome parts, including the c.a. 1,000 nt-long 3′ partial ORF1-RdRp and complete capsid-encoding genomic regions (ORF2/3) of the identified feline noroviruses, were amplified using a 3′RACE semi-nested (sn) PCR method. For the snPCR reactions, the forward primers of the two screening primer sets (NoV-KDEL-Fgen for PCR1 and JV12Y-RW for PCR2 reactions) were used (Table 1).

A total of six of the eight feline norovirus positive samples were 3′RACE-snPCR positive, half of which were already positive in the 1st PCR round. All six positive PCR products (*n* = 3 PCR1 and *n* = 3 PCR2 products) were individually sequenced by NGS with c.a. 1 M read depth. Only a single consensus sequence/sample could be assembled from all six samples. All six consensus sequences showed ≥66.2% nt pairwise identity to each other and 87.9%–93.0% nt identity to feline noroviruses of the GenBank as the closest matches identified by BLASTn analyses.

**Fig 3:**
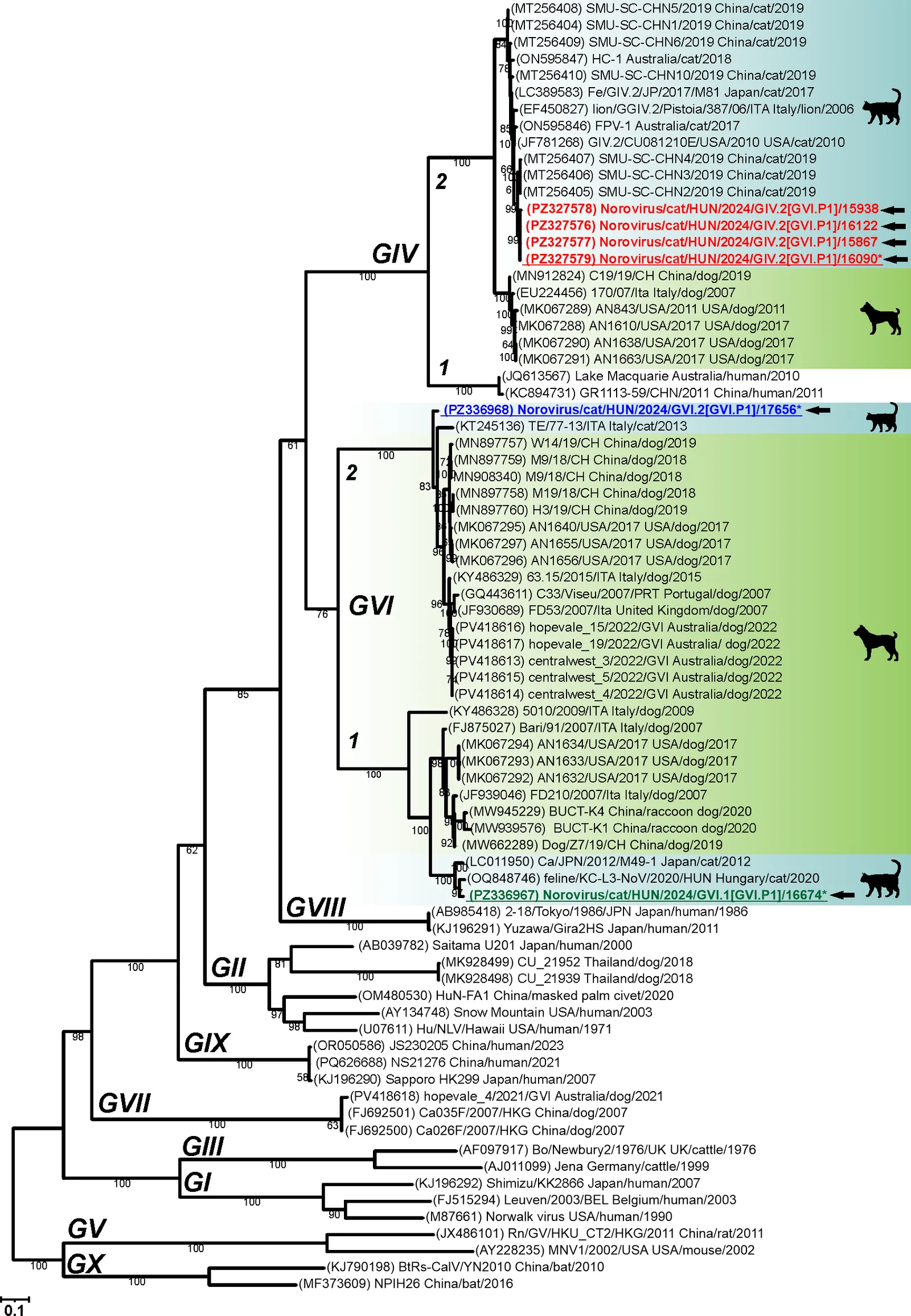
Phylogenetic analysis of complete VP1 amino acid sequences of the study strains (marked with black arrows and written in red, blue, and green according to the RdRp lineages L1–L3/genotypes, respectively). Strains used for complete genome determination are marked with asterisks and underlined. The maximum likelihood tree (best-fit model: LG + F + I + G4 with 1,000 ultrafast bootstrap/BS/ replicates) contains all known feline and canine noroviruses with available corresponding sequences, along with representative sequences of 10 norovirus genogroups (GI–GX). Sequence names include (the accession number) followed by the strain name, country/host/collection year, except for the study sequences, where the sample ID number can be found after the dual-typing designations. Feline and canine origin GIV and GVI sequences are marked with teal and green backgrounds, respectively. Only strains with complete VP1 sequences were included in the analysis.

**Fig 4:**
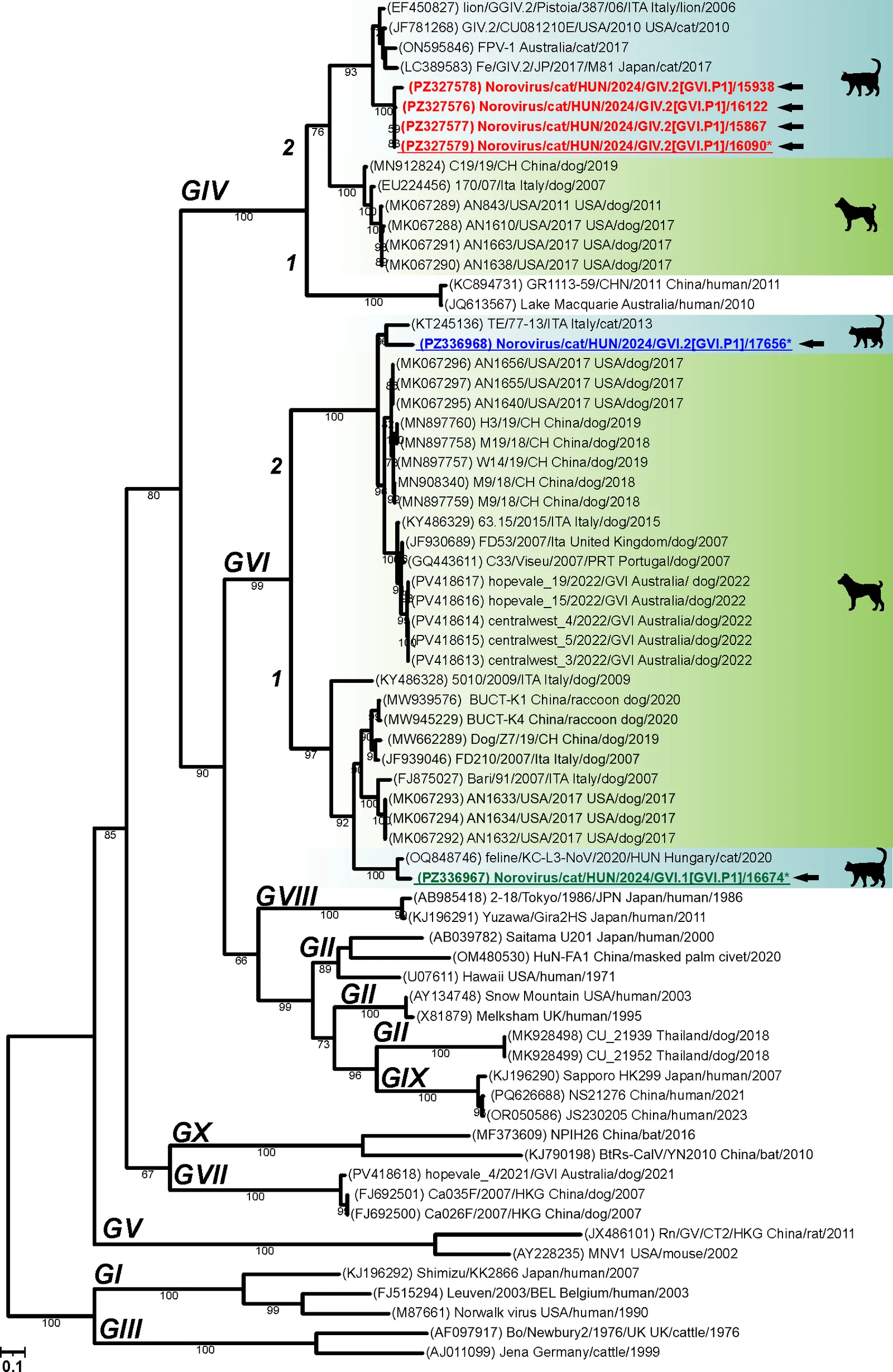
Phylogenetic analysis of complete VP2 amino acid sequences of the study strains (marked with black arrows and labeled in red, blue, and green according to the RdRp lineages L1–L3/genotypes, respectively). Strains used for complete genome sequencing are marked with asterisks and underlined. The maximum likelihood tree (best-fit model: VT + F + I + G4 with 1,000 ultrafast bootstrap/BS/ replicates) contained all known feline and canine noroviruses with available complete VP2 sequences, along with representative sequences of 10 norovirus genogroups (GI–GX). Sequence names include (the accession number) followed by the strain name, country/host/collection year, except for the study sequences, where the sample ID number can be found after the dual-typing designations. Feline- and canine-origin GIV and GVI norovirus sequences are marked with teal and green backgrounds, respectively.

The results of the VP1 amino acid-based phylogenetic analysis indicate that the six consensus sequences originated from feline noroviruses belonging to two genogroups (GIV and GVI) and within these three genotypes (*n* = 4 GIV.2, *n* = 1 GVI.1, and *n* = 1 GVI.2) (Fig. 3). The six study feline norovirus strains show the same clustering in the VP2-based phylogenetic tree, as well (Fig. 4).

The VP1 and VP2 sequences of the four GIV.2 strains (sample IDs: 15867, 15938, 16090, and 16122) are highly similar to each other (≥99.8% and ≥98.6% pairwise nt identity, respectively) and clustered together to form lineages distinct from the other known GIV.2 feline norovirus strains (Fig. 3 and 4).

The VP1 and VP2 sequences of the GVI.1 study strain (sample ID: 16674) exhibited the highest nucleotide identities (94.6% and 92.8%, respectively) and closest phylogenetic relationship with the Hungarian feline norovirus strain, feline/KC-L3-NoV/2020/HUN (OQ848746) (Fig. 3 and 4).

Interestingly, BLASTn analysis showed that the VP1- and VP2-encoding regions of the feline GVI.2 norovirus strain 17656 were more closely related to a canine norovirus strain dog/GVI.2/AN1640/USA/2017 (MK067295), sharing 89.5% and 87.3% nt sequence identity, respectively, than to the other previously reported feline GVI.2 strain from Italy (KT245136), with which they shared only 88.5% and 84.3% identity, respectively, with full query cover.

However, the GVI.2 strain in this study was phylogenetically distinct from all feline and canine GVI.2 noroviruses in VP1 but clustered with the feline GVI.2 strain in the VP2 phylogenetic tree (Fig. 3 and 4).

The 3′ genome parts of the strains contained between one and eight individual SNP sites: two strains had one SNP residue each, while one strain each contained two, four, five, or eight SNPs. Most SNP sites were located in the VP1-encoding ORF2 (*n* = 11) and the VP2-encoding ORF3 (*n* = 8) regions, while only *n* = 2 SNP sites were found in the RdRp-encoding ORF1 region (Fig. S1).

In summary, the results of RdRp-, ORF1-, and capsid-based phylogenetic typing showed that all eight feline norovirus strains shared the same *P* type (GVI.P1), and six of them could be characterized further as belonging to three different genotypes (GIV.2, GVI.1, and GVI.2), creating three genome constellations: GIV.2[GVI.P1] (*n* = 4), GVI.1[GVI.P1] (*n* = 1), and GVI.2[GVI.P1] (*n* = 1). The three lineages identified in the RdRp phylogenetic trees could correspond to the three different genome constellations L1: GIV.2[GVI.P1], L2: GVI.2[GVI.P1], and L3: GVI.1[GVI.P1] (Fig. 1 to 4). The host-based phylogenetic separation of all feline and canine strains was clearly observable in all trees (Fig. 1 to 4).

### Background information on the feline norovirus RT-PCR-positive animals

**Fig 5:**
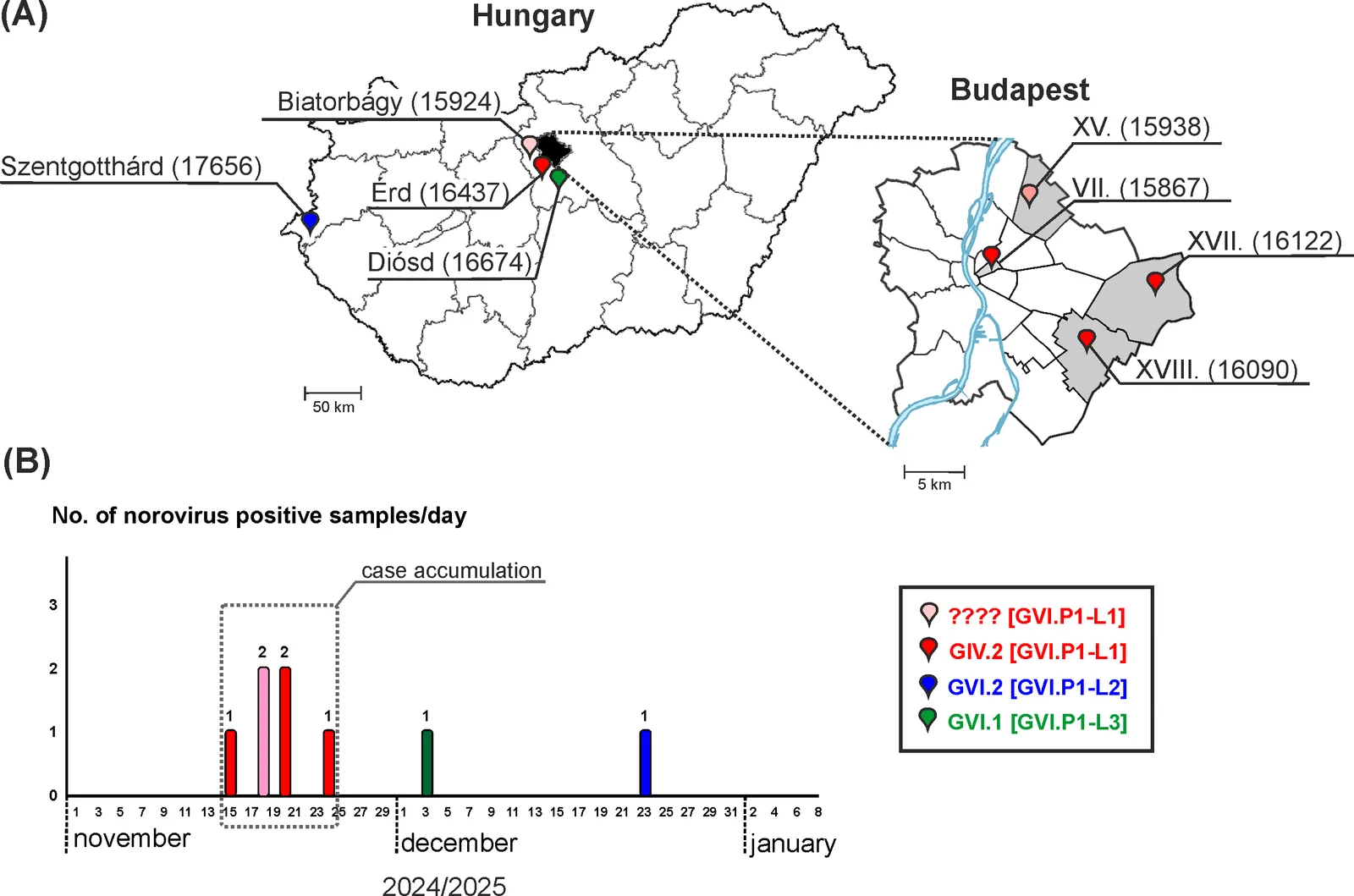
(**A**) Geographic localizations of the sample collection sites (town names or capital districts in roman numbers followed by sample IDs in brackets) together with (**B**) a diagram of the collection dates of the positive samples. The color legends of the pins on the map and the bars on the diagram can be found in the Fig. insert. L1, L2, and L3: Lineages found in the RdRp tree (Fig. 1).

All the positive samples were collected from diarrheic cats aged between approximately 2 and >12 months, which previously had additional diarrhea-associated microbiological tests as well. Only two of the eight affected cats had positive test results for *Giardia* and/or *Toxocara* (Table 2). All GIV.2[GVI.P1] and GVI.P1-L1 norovirus-positive samples were collected during an apparent case accumulation between 15 and 24 November 2024 from geographically close locations: different districts (VII, XV, XVII, XVIII) of Budapest and its suburbs (Érd and Biatorbágy). All except one of the GIV.2[GVI.P1] and/or GVI.P1-L1-positive animals were freshly captured stray cats, all of which had signs of diarrhea at the time of capture (Fig. 5; Table 2). The remaining two samples were collected later from indoor cats with continuous outdoor access living in more distant locations (GVI.1[GVI.P1-L3] from Diósd on 3 December and GVI.2[GVI.P1-L2] from Szentgotthárd on 23 December) (Fig. 5).

**TABLE 2:** Summary of background information and additional microbiological test results for the sampled cats^*a*^

Sample IDs	Identified NoV type	Clinical signs	Housing conditions	Age	Additional microbiological test results
15,867	GIV.2[GVI.P1]	Bloody diarrhea	Stray (freshly captured)	>12 mo	***Giardia* cysts pos**.; helminth egg neg., *Salmonella* sp. and *Campylobacter* sp. neg.
15,924	???? [GVI.P1]	Diarrhea	Indoor (free-roaming)	≈5 mo	Helminth egg neg., protozoon neg.; *Salmonella* sp. and *Campylobacter* sp. neg.
15,938	GIV.2[GVI.P1]	Diarrhea	Stray (freshly captured)	>12 mo	Helminth egg neg., protozoon neg.; *Salmonella* sp. and *Campylobacter* sp. neg.
16,090	GIV.2[GVI.P1]	Diarrhea	Stray (freshly captured)	>12 mo	Helminth egg neg., protozoon neg., *Salmonella* sp. and *Campylobacter* sp. neg.
16,122	GIV.2[GVI.P1]	Diarrhea	Stray (freshly captured)	>12 mo	***Giardia* cysts pos.; *Toxocara* eggs pos**.; *Salmonella* sp. and *Campylobacter* sp. neg.
16,437	???? [GVI.P1]	Diarrhea	Stray (freshly captured)	<6 mo	Helminth egg neg., protozoon neg.; *Salmonella* sp. and *Campylobacter* sp. neg.
16,674	GVI.1[GVI.P1]	Diarrhea (recurrent)	Indoor (free-roaming)	≈8 mo	Helminth egg neg., protzoon neg.; *Salmonella* sp. and *Campylobacter* sp. neg.
17,656	GVI.2[GVI.P1]	Diarrhea	indoor (free-roaming)	≈2 mo	Helminth egg neg., protzoon neg.; *Salmonella* sp. and *Campylobacter* sp. neg.

^*a*^ Positive test results are written in **bold**. Note that the ages of stray cats were based on rough estimation (body size, teeth type, tartar...).

### Pairwise genetic distance analyses of feline and canine GVI.P1&2 noroviruses

**Fig 6:**
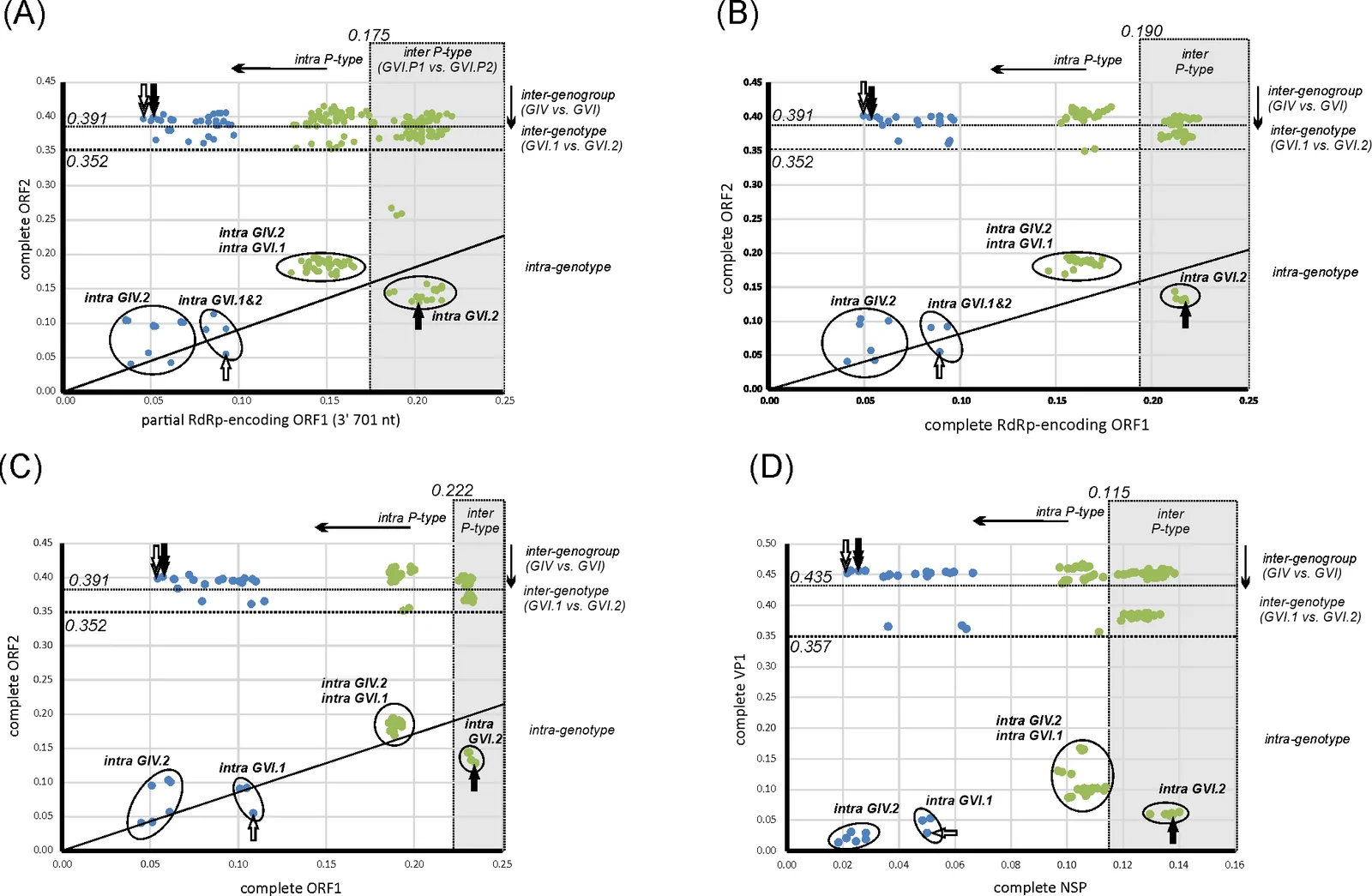
Pairwise distance (p-distance) comparison (PDC) plots between 3′ partial (701 nt) RdRp and complete ORF2 (**A**), between complete RdRp and ORF2 (**B**), and between complete ORF1 and ORF2 (**C**), as well as between the complete non-structural protein (NSP) and VP1 protein (**D**), of feline and canine [GVI.P1 and 2] norovirus sequence pairs, including study strains. The positions of the p-distance points (colored circles) are based on the p-distances of partial (**A**) or complete (**B**) RdRp or complete ORF1 (**C**) (*x*-axis) and the p-distances of ORF2/VP1 (*y*-axis) of the viral pairs. Blue circles represent feline-feline, whereas green circles represent feline-canine norovirus sequence comparisons (note that, for the sake of clarity, the canine-canine norovirus p-distance points are not included in this figure but can be seen in the supplemental tables, Tables S1 to S4). Diagonal lines (**A–C**) are linear regression lines calculated from the p-distance points of virus pairs of the same host (cat or dog), members of which both belong to the same genotype and P-type. Horizontal dotted lines indicate the lowest ORF2 p-distances found between viruses of different genogroups (GVI vs GIV, thinner lines) or between different genotypes (GVI.1 vs GVI.2, thicker lines). Vertical dotted lines indicate the lowest RdRp/ORF1 p-distances found between virus pairs of different P-types (GVI.P1 vs GVI.P2). Inter-P-type p-distance regions are marked with a gray background. P-distance points originating from the comparison of potential recombinant norovirus strains (GVI.2[GVI.P1]PZ336968|17656| cat and GVI.1[GVI.P1]OQ848746|KCL3|cat) with possible ORF2 donors (black and empty simple arrows) and possible ORF1 donors (black and empty double arrows) are also marked. For the original diagrams, the linear regression lines and p-distance data are presented in Tables S1 to S4.

To investigate the relationship of genetic distances between non-structural (complete/partial ORF1/NSP) and structural (complete ORF2/VP1) genome regions of both newly acquired and publicly available domestic feline and canine [GVI.P1 and 2] norovirus sequences, various pairwise distance (p-distance) comparison (PDC) plots were generated (Fig. 6A through D; Tables S1 to S4). In these plots, the p-distance points are formed in multiple cloud-like groups, which can correspond to different intra- and inter-genotype/P-type and inter-genogroup sequence comparisons between feline only or between feline and canine noroviruses (Fig. 6A through D). Inter-genogroup and inter-genotype p-distance points are most clearly separated in complete ORF1/ORF2 nt (Fig. 6C) and complete NSP/VP1 aa PDC plots (Fig. 6D), where the lowest nt/aa ORF2/VP1 p-distances between viruses of different genogroups (GVI vs GIV pairs) or between different genotypes (GVI.1 vs GVI.2 pairs) are 0.391/0.435 and 0.352/0.357, respectively (Fig. 6C and D). While the inter P-type (GVI.P1 vs. GVI.P2 pairs) p-distance points are the most clearly separated in complete RdRp/ORF2 and complete ORF1/ORF2 nt PDCs, where the lowest RdRp/ORF1 nt p-distances are 0.190/0.222, respectively (Fig. 6B and C). The p-distance points of feline-feline virus pairs are also clearly separated from the feline-canine pairs, even when both members of the pair belong to the same genotype and/or P-type. These p-distance points from inter-host virus pairs are located between the levels of inter-genotype/P-type p-distances and the intra-host p-distance points, both in nt and aa PDC plots (Fig. 6A through D).

### Analysis of host-specific amino acid substitutions in the non-structural and capsid proteins

To investigate the potential reasons behind the host-based separations observed in the PDC plots and phylogenetic trees, the distributions of host-specific aa substitutions between feline and canine noroviruses were analyzed. For these analyses, the complete non-structural polyprotein (NSP) of all [GVI.P1] (regardless of capsid type) and complete VP1 and VP2 capsid sequences of GIV.2, GVI.1, and GVI.2 (regardless of the P type) domestic feline and canine noroviruses were aligned separately, and positions with host-specific amino acids (set of residues that are different between cat and dog noroviruses) were highlighted and counted.

**Fig 7:**
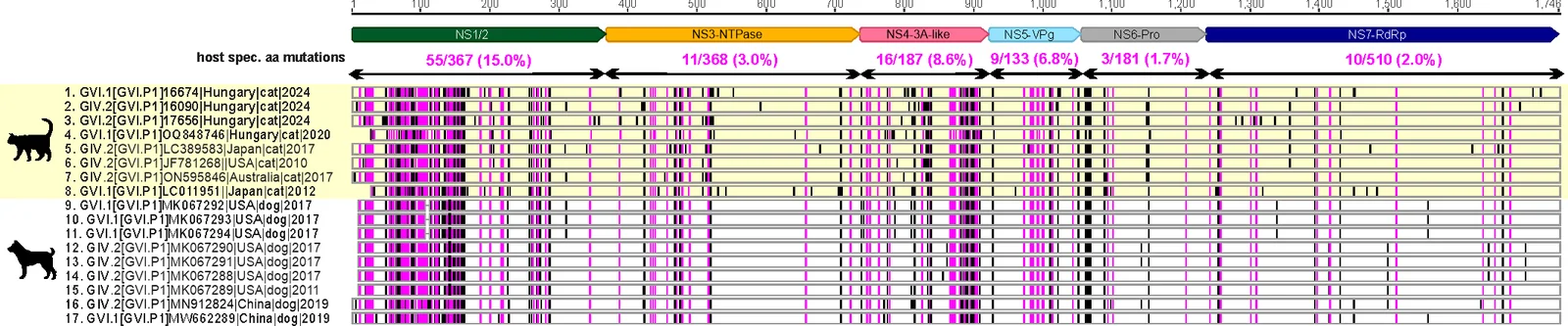
Localizations of host-specific (domestic cat/dog) amino acid (aa) substitutions (magenta vertical lines) in the alignment of complete non-structural polyproteins of all known feline (with pale yellow background) and canine [GVI.P1] noroviruses (bold font indicates study strains). The positions of the non-host-specific variable aa-s are indicated by black vertical lines. A semantic norovirus genome map with the localizations of the main non-structural viral peptides (colored) is shown above the alignment. The absolute no. of host-specific aa mutations/total length (in percentages) of the different peptides were found under the genome map. A more detailed version of this aa alignment with the localizations of host-specific residues can be found in the Supplemental material (Fig. S2).

A total of 104 (6.0%) host-specific aa substitutions were found in the 1,746 aa-long NSP, which are mostly located on the NS1/2 (15.0%), NS4 (8.6%), and NS5 (6.8%) regions, whereas the viral enzymes contained a considerably lower number of mutations (NS3-NTPase: 3.0%, NS6-protease: 1.7%, and NS7-RdRp: 2.0%) (Fig. 7; Fig. S2).

**Fig 8:**
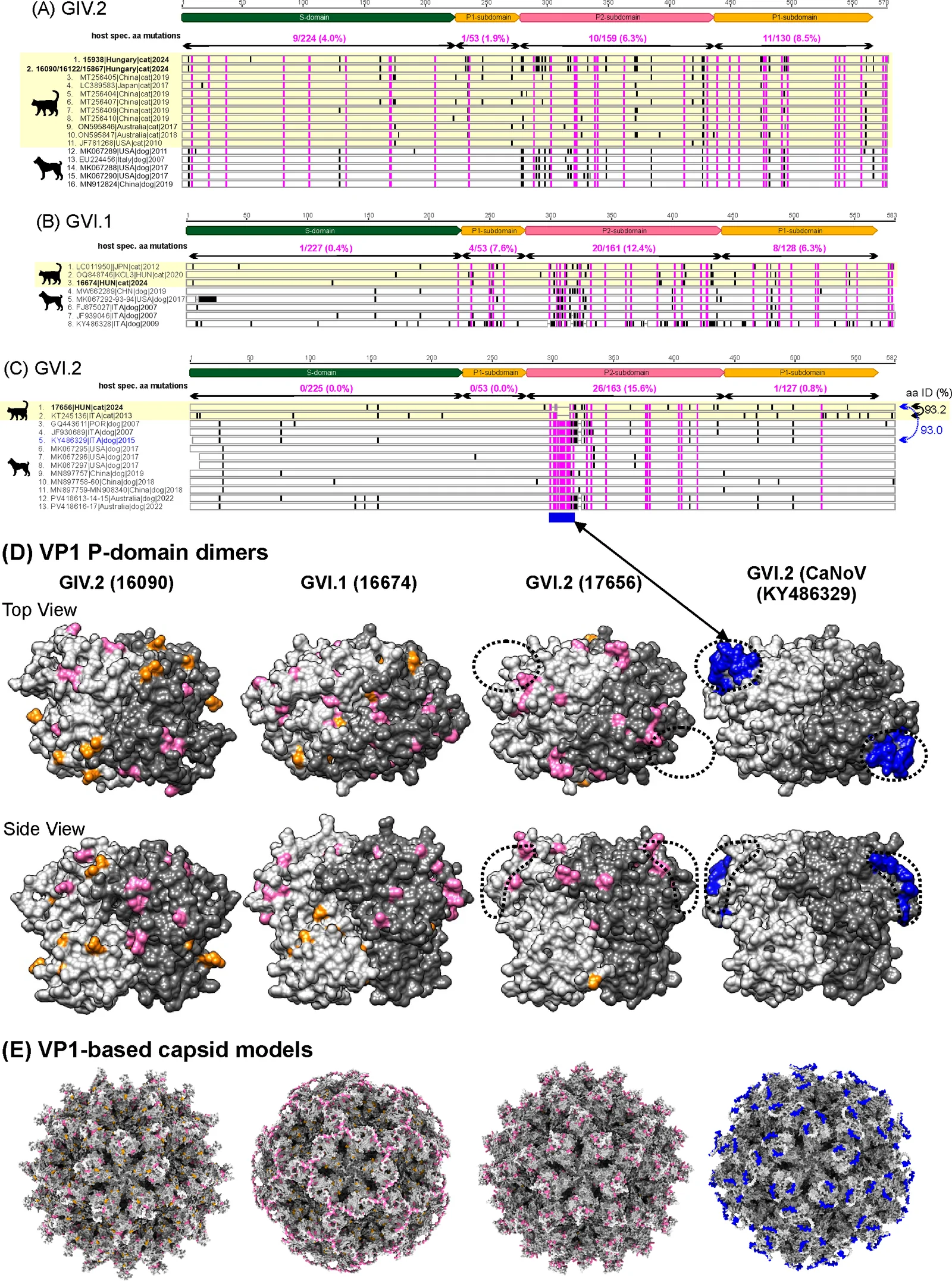
Localizations of host-specific (domestic cat/dog) amino acid (aa) substitutions (magenta vertical lines) in the alignments of complete VP1 proteins of all known feline (with pale yellow background) and canine GIV.2 (**A**), GVI.1 (**B**) and GVI.2 (**C**) noroviruses (study strains in **bold**). Only one of the identical sequences is shown in the alignment, and its name contains the identifiers of the other identical sequences. The positions of non-host-specific variable aa-s are marked with black vertical lines. A semantic map with the localizations of the main VP1 domains/subdomains (colored) is located above the alignments. The absolute no. of host-specific aa mutations/total length (in percentages) of the different domains/subdomains were found under the map. The aa identities (aa ID) between the complete VP1 protein of the study GVI.2 strain (17656) and the most closely related feline (black arrow) and canine (dotted blue arrow) strains are shown on the right side of panel C. A more detailed version of the alignments with the domain/subdomain borders and localizations of host-specific aa substitutions can be found in the Supplemental material (Fig. S3). Three-dimensional models of the VP1 P-domain dimers (**D**) and complete VP1-based capsids (**E**) of the three GIV.2, (16090) GVI.1 (16674), and GVI.2 (17676) feline norovirus study strains together with a canine GVI.2 strain (KY48632). The P-domain monomers are colored pale and dark gray. All four P-domain dimer models were aligned using the MatchMaker function of Chimera and separated manually to maintain identical orientations. Surface-exposed host-specific aas in the feline norovirus P-domain and capsid models are colored according to their localizations (P1-/orange or P2 sub-domains/pink; see panels A–C). In the GVI.2 canine norovirus model, the blue regions indicate P2 domain extensions/bulges (vertical blue line in panel C) that are missing in the feline GVI.2 study strain (the positions are marked with dotted line circles on the P-domain dimer model).

In the case of VP1 capsid comparisons, 31/566 (5.5%), 33/569 (5.8%), and 27/568 (4.8%) host-specific aa substitutions were found among the GIV.2, GVI.1, and GVI.2 noroviruses, respectively, and most of them are located on the P1/P2 subdomains (Fig. 8; Fig. S3). Interestingly, while the majority of host-specific substitutions are located on the C-terminal P1 subdomain (8.5%), followed by P2 (6.3%) and S (4.0%) in the GIV.2 noroviruses (Fig. 8A; Fig. S2A), these mutations were mostly concentrated in the P2 subdomain in the GVI.1 (12.4%) and GVI.2 (15.6%) strains (Fig. 8B and C; Fig. S3B and C). In the case of GVI.2 viruses, in addition to host-specific substitutions of the P2 subdomain, only a single additional mutation was found in the entire VP1 between canine and feline strains (Fig. 8C; Fig. S3C). It is also interesting to note that there is only one additional amino acid difference between the study GVI.2 strain (17656) and its closest relatives: the feline GVI.2 sequence (KT245136; 39/572 amino acid differences, 93.2% aa identity) and a canine GVI.2 sequence (KY486329; 40/578 amino acid differences, 93.0% aa identity) (Fig. 8C; Fig. S3C).

To investigate the localization/surface exposure of these host-specific aa-s in the capsid, multiple three-dimensional models of the VP1 P-domain dimers were generated (Fig. 8D), and VP1-based capsids (Fig. 8E) of the three GIV.2 (16090), GVI.1 (16674), and GVI.2 (17676) strains were modeled, together with a canine GVI.2 strain (KY48632, which is the most closely related strain to the study 17,656 GVI.2 feline norovirus sequence). Most of the identified host-specific residues (27/31 [87.1%], 24/33 [72.7%], and 23/27 [85.2%]) are located on the surfaces of the GIV.2, GVI.1, and GVI.2 models, respectively (Fig. 8D and E). Notably, the P2-subdomains of both GVI.2 feline noroviruses (the study strain [17656] and strain from Italy [KT245136]) contain 10- and 12-aa-long deletions in the same position, respectively, compared with the canine GVI.2 strains (Fig. 8D and E; Fig. S3C). The affected region forms a well-visible bulge on the surface of the P-domain dimers as well as on the surface of the capsid model of GVI.2 canine norovirus; this bulge is missing from the surface of the two GVI.2 feline strains (Fig. 8D and E). Note that due to low confidence in the prediction accuracies of the apical regions on the capsid models (Fig. S5), which was mostly due to the considerable divergence of the P-domain sequences between the study strains and the FoldaVirus reference sequence (GII.2), the shapes of the apical capsid structures are unreliable. These capsid models were used only for predicting the surface-associated nature of amino acid motifs/regions.

**Fig 9:**
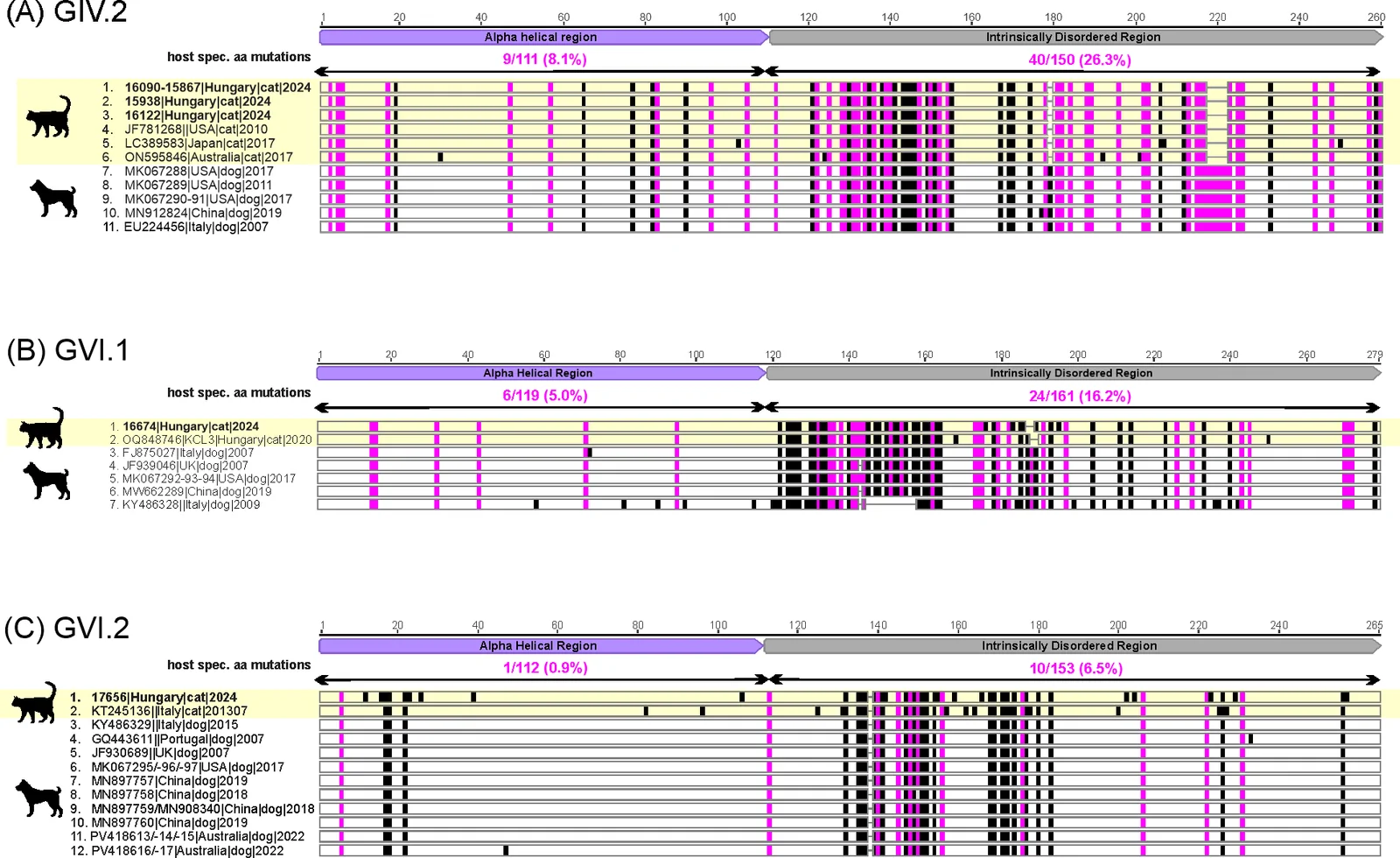
Localizations of host-specific (domestic cat/dog) amino acid (aa) substitutions (magenta vertical lines) in the alignments of complete VP2 proteins of all known feline (with pale yellow background) and canine GIV.2 (**A**), GVI.1 (**B**), and GVI.2 (**C**) noroviruses (study strains in **bold**). Note that only one of the identical sequences is shown in the alignment, and its name contains the identifiers of the other identical sequences. The positions of non-host-specific variable aa-s are marked with black vertical lines. A semantic map with the localizations of the main VP2 regions (by Dave et al. [27]) is located above the alignments. The borders of the VP2 regions were determined using the AlphaFold Server. The absolute no. of host-specific aa mutations/total length (in percentages) of the different regions was found under the maps. A more detailed version of the alignments with borders of the main VP2 regions and localizations of host-specific aa substitutions can be found in the Supplemental material (Fig. S4).

In the case of VP2 capsid comparisons, a total of 49/261 (18.8%), 30/280 (10.7%), and 27/265 (4.2%) host-specific aa substitutions were found among the GIV.2, GVI.1, and GVI.2 noroviruses, respectively, and most of them are located on the intrinsically disordered regions (Fig. 9; Fig. S4).

### Identification of potential recombinant feline norovirus strains using PDC plots

PDC plots can also be used to detect potential recombinant strains. Based on visible deviations of p-distance points from the linear regression lines which are representing the natural correlations between ORF1/RdRp and ORF2 nucleotide distances in apparently non-recombinant strains in the PDC plots, the following two virus pairs were selected to evaluate potential recombination: pair 1: GVI.1[GVI.P1]OQ848746|KCL3|cat vs GVI.1[GVI.P1]PZ336967|16674|cat and pair 2: GVI.2[GVI.P1]PZ336968|17656|cat vs GVI.2[GVI.P2]MN908340|dog, in which the p-distances of ORF1 sequences were higher (0.11 and 0.23, respectively) than those of ORF2 regions (0.06 and 0.13, respectively) (Fig. 6A through C; Table S3). This could indicate that one member of the virus pair is a potential recombinant, while the other is a possible ORF2/minor donor (or its most closely related known sequence).

Potential ORF1/major donors can also be identified in PDC plots by searching for virus pairs in which the potential recombinant shows the lowest p-distance value in ORF1 with another strain, which could be a potential major donor (or its most closely related known sequence).

In our example, GIV.2[GVI.P1]JF781268|cat and GIV.2[GVI.P1]LC389583|cat showed the lowest p-distances (0.05 and 0.06, respectively) in the ORF1 regions, with the potential recombinant sequences of GVI.1[GVI.P1]OQ848746|KCL3|cat and GVI.2[GVI.P1]PZ336968 |17656|cat (Fig. 6A through C; Table S3).

**Fig 10:**
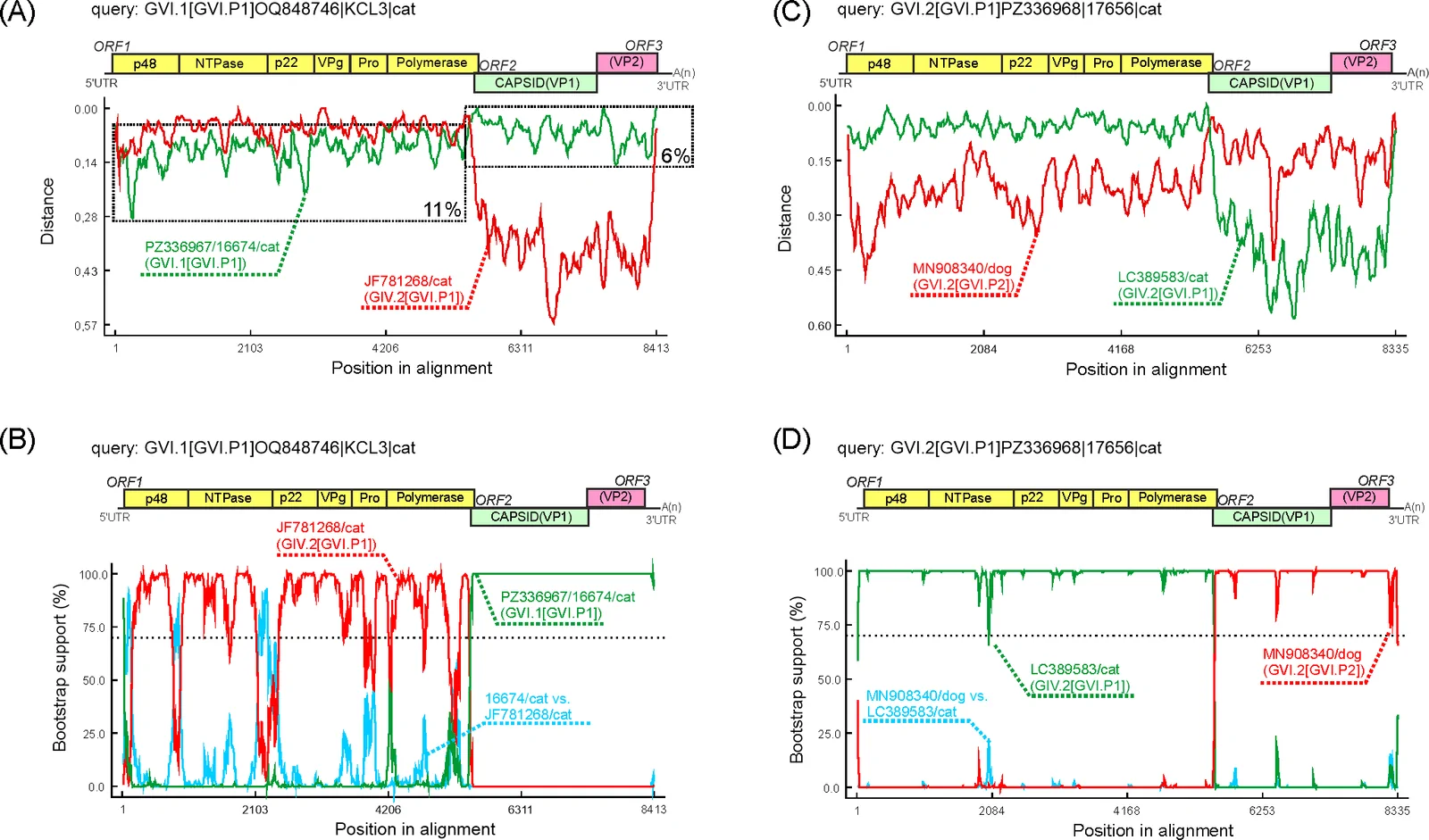
Distance plots (**A and C**) and bootstrap scans (**B and D**) of potential recombinants: GVI.1[GVI.P1]OQ848746|KCL3|cat, (**A and B**) and GVI.2[GVI.P1]PZ336968|17656|cat (**C and D**) used as query sequences. Potential donor sequences are marked with green and red lines, while blue lines represent outgroup sequences with designations (Acc. Num. /host and dual typing). The cut-off percentage was 70 (dotted lines in panels B and D). Semantic norovirus genome maps are shown above the plots to aid orientation. The percentage data displayed in the dotted line boxes in panel A represent the average distance percentages between the corresponding genome parts of the query and subject sequences.

The distance plot and bootscan analyses of these two potential recombinant strains (GVI.1[GVI.P1]OQ848746|KCL3|cat and GVI.2[GVI.P1]PZ336968|17656|cat) and their donor sequences identified by PDC plots also support the recombinant nature of these viruses, with possible recombination breakpoints located on the ORF1/ORF2 junctions (Fig. 10B and D).

Note that because of the relatively low sequence difference (only 9%) in the ORF1 region between the two potential donor sequences of GVI.1[GVI.P1]OQ848746|KCL3|cat, the bootstrap support of the ORF1 donor is not uniform (Fig. 10B), indicating that the selected virus is most likely not the true donor but only the most closely related available sequence.

## DISCUSSION

In our retrospective investigation, the norovirus detection rate (8.9%) was higher than previously reported in the literature (e.g., 6.2% [18], 4.6% [21], and 1.2% [28]). This difference may partly reflect variation in sampling periods (winter months in our cases vs whole year or spring-summer in the previous studies). However, because the sampled animals represented heterogeneous populations, the observed detection rate should not be interpreted as prevalence data.

Although previous studies have indicated that noroviruses are true enteric pathogens in cats (16, 17, 22), in our cases, the definitive correlation between norovirus infections and diarrhea is only suspected because all the positive animals were either stray or free-roaming cats. These animals may harbor several undetectable or uninvestigated co-infecting pathogens (in addition to the *Toxocara* and/or *Giardia* infections identified in two animals), which could also be associated with the manifested enteric symptoms (29).

Using a previously reported 3′RACE semi-nested PCR coupled NGS-based norovirus detection pipeline (30), followed by phylogenetic and sequence analyses, all of the known feline norovirus genotypes (GIV.2 [25], GVI.1 [22], and GVI.2 [18]) were identified in six of the eight positive samples, with no observable genotype/variant co-infections and low intrahost single-nucleotide variations. Two of the three genotypes (GIV.2 and GVI.2) were reported for the first time in Hungary. This substantial number of co-circulating genotypes found in only a few samples strongly suggests that the true genotype diversity of cats could be underestimated. Unfortunately, the majority of the previous studies contained no information about the genotype diversity of the detected feline norovirus strains; therefore, this assumption cannot be validated by literature data (18, 21, 28).

Notably, unlike canine noroviruses, which share two GVI P-types (P1 and P2), all detected capsid types in our cat strains were found in conjunction with a single P-type [GVI.P1], which is also universally present in all other known feline noroviruses, regardless of genotype. This strongly indicates successful host adaptation and the necessity of this polymerase type for the long-term survival and spread of the virus among feline hosts, as previously suggested (31). The identified GVI.P1 lineages (GVI.P1-L1-L3) are correlated with different capsid types, suggesting divergent evolutionary pathways for different genotypes (23).

Analyses of various PDC plots have shown that the most reliable genome regions for sequence-based typing of feline and canine GVI and GIV noroviruses are either the nucleotide sequences of the complete RdRp-encoding region or the complete ORF1 itself for P-typing, and the commonly used complete VP1 amino acid sequences for genotyping (10). The determined lower boundaries for inter-genogroup, inter-genotype, and inter-P-type p-distances fell within the ranges reported by other studies (40%–60% and 20%–40% as well as <30%, respectively) (23, 31) and may be used as lower cut-off values for sequence-based genotype/P-type demarcation of carnivore GIV and GVI noroviruses. The requirement for a complete RdRp/ORF1 region for accurate sequence-based P-type determination underscores the critical importance of longer, ideally full-length, genome sequences in comprehensive norovirus genomic analyses (23). In contrast, our results show that the p-distances of partial RdRp sequences lack sufficient resolution and are therefore inadequate for reliable sequence-based P-typing.

Among the identified genotypes, GIV.2 was predominant, whereas the other two were only sporadically detectable (one of each). At the time of manuscript preparation, GIV.2 also comprised the majority of feline norovirus capsid sequences in GenBank (12/15, 80%) from various countries worldwide, suggesting the possible predominance of this genotype in domestic cats (15). Nevertheless, the low enteric prevalence (1.2%) and low seroprevalence (6.6%) of GIV.2 compared with GVI (26.2%) reported among Japanese cats suggest that genotype dominance may be subject to significant geographic or temporal variations, which should also be investigated (20, 28). These observations are based on limited and potentially biased sequence and prevalence data; therefore, broader and geographically representative epidemiological studies are required to assess the true prevalence and distribution of these genotypes in cats.

Furthermore, only genotype GIV.2 has been reported to be associated with acute gastroenteric outbreaks among cats (and also among dogs), although only in enclosed settings such as animal shelters (17, 32). In our case, the retrospectively identified clonal spread of nearly identical GIV.2[GVI.P1-L1] and [GVI.P1-L1] strains (the latter with undeterminable capsids, most likely due to low viral loads) suggests the presence of a past case accumulation as part of a potential outbreak caused by this genotype confined to a well-defined geographic area (in and around the capital Budapest) in November 2024.

The identified single GVI.1 study norovirus strain (ID:16,674) is most closely related to a GVI.1[GVI.P1] strain (feline/KC-L3-NoV/2020/HUN, OQ848746), which was identified from a diarrheic stray kitten in 2020, also from Hungary. The detection of this genotype in both 2020 and 2024 suggests that it is most likely endemically present and continuously circulating among Hungarian cat populations. In addition to the two Hungarian strains, only a single Japanese GVI.1 feline norovirus strain has been identified; therefore, the global prevalence of this genotype is largely unknown.

PDC plots of the ORF1/ORF2 genome regions can help identify potential recombinant sequences, as previously described (11). The discrepancies in the ORF1 and ORF2 p-distances could indicate a past recombination event affecting the ORF1/ORF2 junction of the ancestor of feline/KC-L3-NoV/2020/HUN, which was also supported by the results of bootscan and RDP analyses. ORF1/ORF2 junctions as recombination hotspots have also been reported in other noroviruses (11, 33).

Strain 17656 is the second reported feline GVI.2 norovirus, after the partial Italian strain TE/77-13/ITA (KT245136) (18), and the first with a complete genome. The results of PDC, bootscan, and distance plot analyses suggest that strain 17656 is an ORF1/ORF2 recombinant, similar to the previously reported feline GVI.2 strain. However, the phylogenetic separation of the two feline strains and the higher sequence identity of strain 17656 to canine GVI.2 viruses suggest that they may have arisen through two independent recombination events between canine and feline noroviruses at the ORF1/ORF2 junction.

The observed co-clustering of VP2 proteins of the two feline GVI.2 strains may be the result of the previously observed covariation between VP1 and VP2 sequences (34), which could be connected with the potential adaptation of canine-origin VP1 to a novel feline host. Moreover, to our knowledge, there is currently no direct experimental evidence demonstrating that canine noroviruses can successfully infect feline hosts or replicate in feline-derived cell lines, and vice versa. This raises the question of the original host of these recombinant strains (not just GVI.2[GVI.P1] but also GIV.2[GVI.P1] strains), which would not necessarily be a dog or cat. A few norovirus sequences have been identified in wild carnivores that are phylogenetically closely related to feline (for example, GIV.2[GVI.P1] strains detected in lions [25]) or canine noroviruses (for example, GVI.1[GVI.P1] strains reported in raccoon dogs [35]). The presence of viruses closely related to domestic feline and canine noroviruses in wildlife suggests that wild carnivores may act as original and/or intermediate hosts in the emergence and adaptation of host-switching recombinant viruses. However, the currently available sequence data from wild carnivores is insufficient to verify this hypothesis.

Nevertheless, the previously suspected cross-species transmission of noroviruses between domestic dogs and cats (17, 18) is likely uncommon, or at least these spillovers do not frequently lead to sustained transmission. This conclusion is supported by the clear host species-based separation of all feline and canine genotypes observed in phylogenetic trees and PDC plots. The degree of host-based separation does not reach the levels of inter-genotype/P-type segregations in either phylogenetic trees or sequence p-distances. This is likely due to the host species adaptation process, which affects all NS and VP proteins but not to the same extent, as well as the accumulation of (non-)synonymous random mutations resulting from the divergent evolution of noroviruses in different hosts, similar to those reported between human and carnivore noroviruses (14). Sequence comparisons showed that the majority of host-specific aa substitutions in NSP were located in the NS1/2 (p48), NS4 (p20), and NS5 (VPg) proteins, which play a pivotal role in interaction with host-specific cellular factors during replication but also have a higher mutation rate than viral enzymes (36). Therefore, the higher mutation rate of these protein-encoding genome parts could mask the true host-specific aa substitutions that are important for successful host adaptation.

Surface-exposed host-specific substitutions *in silico* identified on the capsid may have involved in intermolecular contacts, making these residues significant for functional adaptation and specificity (37). However, their functional significance in immune evasion and/or host-specific binding of receptors and/or attachment factors, such as HBG antigens, which are well-known functions of VP1, requires experimental validations (12, 38, 39).

The 10- or 12-amino-acid deletion found in the highly variable P2 subdomain of feline GVI.2 may alter surface-exposed features of VP1 and could potentially influence cellular attachment or immune recognition (14, 40). Interestingly, a similar deletion pattern was observed between human and carnivore GIV.2 strains as well, further supporting the pivotal role of this region in host-based segregation (14). However, due to the highly variable nature of the P2 subdomain, additional GVI.2 sequences and experimental studies are necessary to confirm the predominance and role of this deletion in the adaptation process or immune evasion of GVI.2 noroviruses in cats.

The highly diverse VP2 minor capsid is a multifunctional peptide characterized by a conserved N-terminal α-helical region and an intrinsically disordered C-terminal region. The peptide has region-specific functions in genome encapsidation by interacting with VP1 and VPg peptides (5, 27). Sequence analyses indicated that the C-terminal region contains most of the host-specific amino acid substitutions in all three genotypes of feline noroviruses and exhibits significant sequence diversity (36). Based on the currently known role of VP2 in virion assembly and stability, most of its known interactions are associated with viral, not host peptides; therefore, the role of these observed host-specific substitutions remains to be determined.

This study has several limitations. The sequence, phylogenetic, and structural analyses were based on the use of a relatively small number (25 partial and seven complete) of known feline norovirus sequences. This limitation is particularly relevant to the analysis of host-specific amino acid substitutions between feline and canine noroviruses, especially within the GVI.1 and GVI.2 genotypes. Consequently, these results should be interpreted as preliminary observations. Furthermore, the hypothesized biological functions of surface-exposed VP1 residues, or deletions also require experimental validation. Additional norovirus sequences from feline and canine hosts, as well as *in vitro* virus attachment studies, are required to validate the results of these *in silico* comparative analyses.

In conclusion, the current understanding of the genetic diversity, prevalence, and epidemic potential of feline noroviruses remains significantly incomplete, primarily because of the limited availability of complete genomic sequences and the lack of large-scale epidemiological studies. The close phylogenetic relationship between small carnivore noroviruses and the presence of extremely rare inter-genogroup recombinants (GIV-GVI) may underscore the significance of small carnivores in understanding the evolution and host adaptation processes of noroviruses.

## MATERIALS AND METHODS

### Background information on samples

Fecal samples were collected from 90 homed pet cats (indoor-kept or free-roaming), stray cats, and shelter cats that showed signs of diarrhea between 1 November 2024 and 31 January 2025. Individual fecal samples were collected from the floorings of the animals with a sterile scoop and stored in fecal specimen containers. Samples were originally sent to a veterinary microbiology laboratory (Duo-Bakt, Veresegyház, Hungary) for routine bacteriological and parasitological testing.

### Nucleic acid isolation and RT-PCR reaction conditions

Approximately 40% (v/v) suspensions were prepared from the fecal samples using sterile 0.1 M phosphate-buffered saline. Suspensions were centrifuged at 10,000 rpm for 5 min, and 150 µL of the supernatant was used for total RNA isolation using the NucleoSpin RNA Kit (Macherey-Nagel, Düren, Germany), according to the manufacturer’s instructions. RNA samples were stored at −80°C until further use.

The conditions and reagents used in the screening and genome acquisition RT-PCR reactions were the same as those described previously, with minor modifications (41, 42). Briefly, in the RT reactions, 2.5 μL of total RNA was added to a final volume of 25 μL, and all RT products were used in PCR reactions. For norovirus screening reactions, two generic primer sets were used, both targeting the ORF1 RdRp-encoding genomic region. The JV12Y-RW/JV13I primers were slightly altered versions (the forward primer contains additional mixed bases) of the broadly used JV12Y/JV13I norovirus screening primers (26), whereas the other novel set (NoV-YGDD-Rgen/NoV-KDEL-Fgen) was newly designed to recognize two conserved aa motifs (YGDD and KDEL) encoding genomic regions of noroviral ORF1 ^RdRp^(Table 1). The PCR products of the two primer sets overlapped; the approximately 550-bp-long NoV-YGDD/-KDEL PCR product contained the target sites of the JV12Y-RW/JV13I primers.

Additional details regarding the oligonucleotide primers used in the screening reactions are listed in (Table 1). For complete ORF1 determination reactions, multiple generic primer sets were designed using nt alignments of GVI.P1 feline noroviruses downloaded from the NCBI GenBank database. The 5′ ends of the genomes were determined using the 5′ rapid amplification of cDNA ends (5′RACE) PCR method, as described previously (42). The primer sequences used for the ORF1 determination reactions are available upon request. The acquired PCR products from screening and ORF1 determination reactions were sequenced directly in both directions using BigDye Terminator v1.1 Cycle Sequencing Kit (Thermo Fisher, Waltham, USA) and ran on an ABI 3500 Genetic Analyzer (Applied Biosystems/Hitachi, Tokyo, Japan). Sequencing data were analyzed using Chromas ver. 2.6.76 (Technelysium, South Brisbane, Australia) and Geneious Prime ver. 2025 (Biomatters, Auckland, New Zealand).

To determine the complete capsid (ORF2/3) and 3′-untranslated regions, a previously described semi-nested 3′RACE PCR (3′RACE-snPCR) method was used (30). Briefly, 5 µL of total RNA was converted to cDNA using an Oligo dT-Anchor-Adapter primer (Table 1). The RT products were digested with RNAse-H (New England Biolabs, Ipswich, MA, USA). Digested cDNA samples were used for semi-nested PCR reactions with a universal adapter reverse primer together with outer (PCR1) and inner (PCR2) generic norovirus forward primers, which were the same as those used in the screening reactions (Table 1). For both PCR rounds, 3 U of Dream Taq polymerase (Thermo Fisher, Waltham, MA, USA) was used in a final volume of 25 µL. The thermal programs of the first and second PCR rounds contained 39 and 26 cycles, respectively. Using this 3′RACE-snPCR method, c.a. 3,644/3,416-bp-long genome regions of the investigated noroviruses could be amplified (Table 1).

### Next-generation sequencing and data analysis

All successful 3′RACE-snPCR products were individually sequenced on a NovaSeq X Plus platform (Illumina, San Diego, CA, USA) and analyzed using an in-house assembled bioinformatics pipeline, as described previously (30). Briefly, a sequencing library was prepared using the xGen DNA EZ Library Prep Kit (IDT, Coralville, IA, USA). Sequencing was performed with a 2 × 151 run configuration and a sequencing depth of 1,000,000 reads for each sample. NGS reads were quality-checked, and adapters were trimmed using fastp 0.21.0v (43). For positive selection of reads, a reference database of noroviruses, including prototype strains from Chhabra et al. (10) and all known feline and canine noroviruses with >3,000 nt long 3′-end genomic sequences, was created. Sequences were downloaded from the NCBI virus database (https://www.ncbi.nlm.nih.gov/labs/virus/vssi/#/). Reads were aligned to the reference database with Local BLAST (blastn 2.15.0+), and only reads that showed > 70% nt identity (<0.1 E-value) to any sequence in the reference database were used for further bioinformatics analyses. Contigs were *de novo* assembled from the positively selected reads using the SPAdes assembler with default settings (44). The generated contigs were searched against a non-redundant database of NCBI using BLASTn. Most similar complete norovirus genomes or complete ORF1^RdRp^-complete ORF2/3 sequences were selected and used as reference sequences in following the reference-guided alignments (Map Reads to Reference). Consensus sequences were generated from the reference-guided alignments using Geneious Mapper (minimum overlap between reads: 50 nt, minimum overlap identity: 95%, maximum mismatch per read: 5%, maximum gap size: 3, word length: 35, index word length: 15) using a 0% threshold option (most common bases in the consensus sequence). Single nucleotide polymorphism (SNP) analyses were conducted on the mapped reads with the “Find SNPs” application of Geneious Prime Ver. 2026.1.2 with the following options: minimum coverage: 5, minimum variant frequency: 0.35, and with other default parameters.

### *In silico* sequence, phylogenetic, recombination analyses, and pairwise distance comparison plots

All available feline (Felidae, ncbitaxon: 9,681, *n* = 25) and canine (Canidae, ncbitaxon: 9,608, *n* = 79) norovirus sequences (ncbitaxon: 142,786), regardless of length, were downloaded from the NCBI GenBank database on 20 January 2026. Strains with only partial ORF2 sequences (*n* = 9) were excluded from further analyses. All norovirus sequences from non-domestic cats and dogs, such as lions, jaguars (*Panthera* sp.), and raccoon dogs (*Nyctereutes* sp.), were used only for phylogenetic analyses, as applicable. Representative genotypes and P-types of all norovirus genogroups/*P* groups (GGI-X), selected from the prototype strain list of Chhabra et al. (10), were also included in the phylogenetic analyses. Multiple sequence alignments of nt and aa sequences were generated using the MUltiple Sequence Comparison by Log-Expectation (MUSCLE) web tool (https://www.ebi.ac.uk/jdispatcher/msa/muscle) with default parameters, except for VP2 aa, for which the MAFFT alignment program was used (https://www.ebi.ac.uk/jdispatcher/msa/mafft). Pairwise sequence identity calculations were performed using the Sequence Identity And Similarity (SIAS) web tool. Phylogenetic analyses were conducted using the IQ-TREE web server (45) with 1,000 ultrafast bootstrap replicates (46) and the best-fit model of substitutions (specified in tree legends). Trees were visualized and annotated using the Interactive Tree of Life (iTOL) v6 (47) and CorelDraw Standard 2020 ver. 22.

All available (GVI. P1 and P2) norovirus sequences of domestic cats or dogs were selected for pairwise distance (p-distance) comparison (PDC) analyses of the nonstructural (ORF1 or RdRp-encoding) and structural (ORF2) genome regions. Genotype and P-type assignments were based on the results of preliminary neighbor-joining-based phylogenetic analyses of MUSCLE-aligned ORF1, RdRp-encoding, and ORF2 nucleotide sequences. The resulting dual genotype/P-type designations were incorporated into the sequence names used in the analyses (Tables S1 to S4). For nucleotide-based PDC analyses, three sequence data sets were compiled: (i) complete or nearly complete ORF1 sequences, with no more than 81 nucleotides missing from the 5′ end, together with their corresponding complete ORF2 sequences (*n* = 26); (ii) complete RdRp-encoding regions of 1,533 nucleotides together with complete ORF2 sequences (*n* = 30); and (iii) at least 700-nucleotide-long sequences from the 3′ end of the RdRp-encoding region together with complete ORF2 sequences (*n* = 42). These data sets included the feline norovirus strains identified in the present study (Tables S1 to S3). In addition, an amino acid-based PDC analysis was performed using complete or nearly complete VP1 sequences, with no more than 27 amino acids missing from the N terminus (Table S4). Separate nucleotide or amino acid alignments were generated for each genomic or protein region. P-distance matrices were calculated in MEGA version 12 using the p-distance method and pairwise deletion of gaps and missing data (48). The distance matrices obtained for the nonstructural regions—complete ORF1, complete RdRp, or the 3′ partial RdRp region—were paired and merged with the corresponding ORF2 distance matrices, and PDC plots were generated in Microsoft Excel for Microsoft 365, version 2601, build 16.0.19628.20166 (Tables S1 to S4). Final PDC plots were prepared for publication using CorelDRAW Standard 2020, version 22.

To detect potential recombinant strains, linear regression lines were fitted in Excel using p-distance points of virus pairs from the same host species that shared both the same genotype and P-type. These regression lines were used as reference baselines representing the natural correlations between ORF1/RdRp and ORF2 nucleotide distances in apparently non-recombinant, host-, genotype-, and P-type-matched virus pairs.

P-distance points deviating from the regression lines were examined as potential indicators of conflicting evolutionary histories between the nonstructural and structural genomic regions. Points located above or below the regression line represented virus pairs with greater or lower ORF2 divergence than their ORF1/RdRp, respectively. Such deviations could be due to the naturally higher substitution rates of the capsid-encoding ORF2 region compared with the NSP-encoding ORF1, or because of a possible historical recombination. The latter is evident in strains where the recombination causes a capsid-type switch, which is the case for all virus pairs whose members belong to different genogroups (GIV vs GVI) or genotypes (GVI.1 vs GVI.2) (Fig. 6A through C).

Potential recombinant strains, together with their putative minor and major parental sequences or their closest available relatives, were further analyzed using Recombination Detection Program version 4.101 (RDP4). Analyses included the automated RDP method, manual BootScan analysis, and distance plots, using the following parameters: model, similarities; window size, 200 nucleotides; step size, 20 nucleotides; 1,000 bootstrap replicates; and bootstrap support cutoff, 70% (49).

### Structural modeling

For structural modeling of P-domain dimers, the Iterative Threading Assembly Refinement (I-TASSER) server (https://zhanglab.ccmb.med.umich.edu/) was used (50). All P-domain dimer models were based on the experimentally determined structure of the feline GIV.2 strain CU081210E (PDB Id: 4QUZ [8]). Models with the highest confidence (C-values) were selected for further analysis. The selected models were visualized and analyzed using Chimera v. 1.20 (51). For VP2 structure prediction, the AlphaFold Server (https://alphafoldserver.com/) was used (52). For the prediction of VP1-based three-dimensional (3D) capsid models, the FoldaVirus capsid prediction web tool (https://foldavirus.org/) was used (53). All *T* = 3 symmetry models were based on the use of the reference structure of a norovirus GII.2 (pbid: 6OTF), which was automatically selected by the FoldaVirus software. The models were visualized and annotated using the ChimeraX ver.1.11.1 software (54). These models were used only for predicting surface-associated amino acid motifs/regions.

## Data Availability

The nucleotide sequence data reported in this study are available in the National Centre for Biotechnology Information Database/GenBank database under the accession numbers PZ327576–PZ327579; PZ327579, PZ327589, PZ336967, PZ336968, and PZ336968.

## SUPPLEMENTAL MATERIAL

Supplemental figures spectrum.01662-26-s0001.docx Fig. S1 to S5.

Table S1 spectrum.01662-26-s0002.xlsx Pairwise distance (p-distance) comparison (PDC) matrix between the nucleotide sequences of the 3′ partial RdRp-encoding ORF1 and complete ORF2 of canine and feline [GVI. P1 and P2] noroviruses.

Table S2 spectrum.01662-26-s0003.xlsx Pairwise distance (p-distance) comparison (PDC) matrix between the nucleotide sequences of the complete RdRp-encoding ORF1 and complete ORF2 of canine and feline [GVI. P1 and P2] noroviruses.

Table S3 spectrum.01662-26-s0004.xlsx Pairwise distance (p-distance) comparison (PDC) matrix between the nucleotide sequences of the complete ORF1 and complete ORF2 of canine and feline [GVI. P1 and P2] noroviruses.

Table S4 spectrum.01662-26-s0005.xlsx Pairwise distance (p-distance) comparison (PDC) matrix between the amino acid sequences of the complete non-structural protein and complete VP1 of canine and feline [GVI. P1 and P2] noroviruses.

ASM does not own the copyrights to Supplemental Material that may be linked to, or accessed through, an article. The authors have granted ASM a non-exclusive, world-wide license to publish the Supplemental Material files. Please contact the corresponding author directly for reuse.
