# Supplementary material for: Feline noroviruses: investigation of epidemic potential and genetic/structural insights of host-based separation and recombination
Source: Microbiol Spectr. 2026 Sep 2;14(10):e01662-26. doi: 10.1128/spectrum.01662-26 (PMC13689052; doi:10.1128/spectrum.01662-26)
Supplement: Supplementary file 1 [file spectrum.01662-26-s0001.docx]

**Supplementary material**

**Feline noroviruses: Investigation of epidemic potential and genetic/structural insights of host-based separation and recombination**

Ákos Boros^a#^, Akari Sugihara^a^, Benigna Balázs ^a^, Fruzsina Tóth^a^, Zoltán Lajos ^b^, Péter Pankovics^a^, Péter Urbán^c^, Róbert Herczeg^c^, Gábor Reuter^a^

^a^	Department of Medical Microbiology, Medical School, University of Pécs, Pécs, Hungary,

^b^	Duo-Bakt Veterinary Microbiology Laboratory, Veresegyház, Hungary

^c^	Szentágothai Research Centre, Bioinformatics Research Group, Genomics and Bioinformatics Core Facility, University of Pécs, Pécs, Hungary.

**Figure S1.:** Localization of small nucleotide polymorphism (SNP) sites (vertical orange bars) of the six NGS-derived feline norovirus consensus sequences (Sequence IDs: left side, colored by genotype). SNPs were identified by Geneious Prime from the alignment of the NGS reads to its corresponding consensus sequences. The localizations of the ORFs (horizontal yellow bars) of the analyzed 3’ norovirus genome region are shown at the top of the figure to help orientation. Ruler: nt positions in alignment.

**Figure S2.**: Localizations of host-specific (domestic cat vs. dog) amino acid (aa) substitutions (aas with magenta background) in the alignment of complete non-structural polyproteins of all known feline (with pale yellow background) and canine [GVI.P1] noroviruses (study strains are the top three viruses). The conserved and non-host-specific variable aa-s are marked with grey and black letters, respectively. A semantic NoV genome map with the localizations of main non-structural viral peptides (colored) is located above the alignment.

**Figure S3.**: Localizations of host-specific (domestic cat vs. dog) amino acid (aa) substitutions (aas with magenta background) in the alignments of complete VP1 proteins of all known feline (with pale yellow background) and canine GIV.2 (A), GVI.1 (B) and GVI.2 (C) noroviruses (study strains in **bold**). The conserved and non-host specific variable aa-s are marked with grey and black letters, respectively. A semantic map with the localizations of the main VP1 domains/subdomains (colored) is located above the alignments.

**Figure S4.**: Localizations of host-specific (domestic cat vs. dog) amino acid (aa) substitutions (aas with magenta background) in the alignments of complete VP2 proteins of all known feline (with pale yellow background) and canine GIV.2 (A), GVI.1 (B), and GVI.2 (C) noroviruses (study strains in **bold**). The conserved and non-host specific variable aa-s are marked with grey and black letters, respectively. A semantic map with the localizations of the main VP2 regions is located above the alignments. The borders of VP2 regions were determined using the AlphaFold Server.

**Figure S5:** FoldaVirus-predicted VP1-based 3D capsid models of the study feline noroviruses (A-C) and a canine GVI.2 strain (D). The models were colored according to the confidence levels of the predictions. Color-coded confidence intervals (pLDDT) determined by the FoldaVirus software are shown at the bottom of the figure. All predictions were based on the use of the reference structure of a norovirus GII.2 (pbid: 6OTF), which was automatically selected by the FoldaVirus software.
